# 2464. Implementation of a *Candida auris* admission surveillance protocol at the NIH Clinical Center

**DOI:** 10.1093/ofid/ofad500.2082

**Published:** 2023-11-27

**Authors:** LaToya A Forrester, Robin T Odom, Angela V Michelin, Joseph Scaletta, David K Henderson, Tara Palmore

**Affiliations:** National Institutes of Health Clinical Center, Bethesda, Maryland; NIH Clinical Center, Jefferson, Maryland; NIH, Bethesda, MD; National Institutes of Health Clinical Center, Bethesda, Maryland; NIH, Bethesda, MD; George Washington School of Medicine & Health Sciences, DC

## Abstract

**Background:**

*Candida auris* (*C. auris*) is a highly transmissible and usually multi-drug resistant yeast that causes serious invasive infections, with a high mortality rate, colonizes patients, and persists in the environment, resulting in outbreaks in healthcare settings. In 04/2019, The NIH Clinical Center (CC) identified a sensitive C. *auris* isolate from a blood culture. In 08/2019, the CC’s first case of resistant *C. auris* was identified from a patient recently hospitalized abroad. By the end of 2019, a total of 17 cases *of C auris* were identified in the state in which our institution resides (Maryland), as well as cases in institutions in the District of Columbia and Virginia (DMV). This led the CC to implement a *C. auris* admission surveillance program.

**Methods:**

From 11/2019 to 03/2023, nares, axilla, throat, and groin swabs (n=1408) were collected on admission from patients hospitalized: 1) abroad, 2) in long-term care facilities (LTAC), or 3) in the DMV within the last 6 months. 467 unique patients were tested, 315 by culture utilizing Sabouraud dulcitol broth containing 10% NaCl, Gentamicin, and Chloramphenicol, incubated at 40 degrees and *Candida* chrome incubated at 30 degrees. 152 patients were tested by PCR, utilizing E-swabs to collect axilla/ groin specimens and aluminum shafted culturette swabs for the nares. PCR testing was conducted at a reference lab from 10/2021 to 07/2022, while routine culturing was unavailable at our facility.

**Results:**

Of 1408 cultures and PCRs collected on admission, no additional patients were identified with *C. auris*.

**Conclusion:**

*C. auris* surveillance is a vital strategy to identify colonized or infected patients. Despite the CC serving a large international patient population and patients hospitalized in facilities from states with a high prevalence of C. *auris*, our surveillance program has not identified additional positives. With cases steadily increasing across the United States (2022 – 2,377 clinical cases and 5,754 screening cases from 29 states) and because of the vulnerability of our patient population, we will continue screening.

**Disclosures:**

**Tara Palmore, MD**, Abbvie: Grant/Research Support|Gilead: Grant/Research Support|Rigel: Grant/Research Support

